# Long-term outcomes of surgical interventions for stress urinary incontinence: a systematic review and network meta-analysis

**DOI:** 10.1097/JS9.0000000000000828

**Published:** 2023-11-02

**Authors:** Yuanzhuo Chen, Chi Zhang, Shiqin Yang, Jiawei Chen, Liao Peng, Jie Chen, Hong Shen, Deyi Luo

**Affiliations:** a Department of Urology, Institute of Urology; b Department of Laboratory Medicine, West China Hospital; c Pelvic Floor Diseases Center, West China Tianfu Hospital, Sichuan University, Sichuan, People’s Republic of China

**Keywords:** long-term, network meta-analysis, stress urinary incontinence, surgical treatment, systematic review

## Abstract

**Background:**

Stress urinary incontinence is common among women, and surgical interventions have significantly improved patients’ symptoms. The long-term effectiveness of these surgeries is increasingly drawing attention, yet it remains sparsely documented in the literature.

**Objective:**

To compare the long-term effectiveness and safety of retropubic tension-free vaginal tape (TVT-RP), tension-free vaginal tape-obturator (TVT-O), transobturator tape (TOT), single-incision sling (SIS), Burch colposuspension, and pubovaginal sling (PVS).

**Methods:**

A comprehensive and systematic literature review was conducted in PubMed, EMBASE, MEDLINE, Cochrane Library, Medicine, and clinicaltrials.gov from inception to May 2023. Selected trials were evaluated for potential bias using the Cochrane tool. Treatment modalities were compared using network meta-analysis to assess objective success rate, subjective success rate, and complications as outcomes.

**Results:**

A total of 37 studies involving 5720 patients were included. No significant statistical differences were found among the interventions regarding objective success rate. PVS had the highest surface under the cumulative ranking curve SUCRA value (93.1). For subjective success rate, TVT-RP, TVT-O, and PVS demonstrated superiority over SIS, with PVS having the highest SUCRA value (80.1). SIS had lower overall complication and pain rates compared to other methods, with statistical significance. There were no differences in reoperation rate, exposure rate, and urinary tract infection occurrence among the surgical approaches.

**Conclusions:**

In terms of long-term effectiveness and safety, TVT-RP and TVT-O appear to be the preferred options for patients opting for synthetic slings, while for patients seeking nonsynthetic slings, PVS may represent the optimal choice.

## Introduction

Highlights

- This study applied a frequency-based network meta-analysis method to analyze data from 37 randomized controlled trials, comparing the long-term effectiveness and safety of different surgical methods for stress urinary incontinence, which has not been reported in previous studies.
- This study, with a median follow-up of 48 months, sheds light on the long-term effectiveness of surgeries for stress urinary incontinence, offering supplementary evidence for comparing surgical outcomes.
- Retropubic tension-free vaginal tape (TVT-RP) and tension-free vaginal tape-obturator (TVT-O) are favorable options for patients willing to undergo synthetic sling placement, while autologous pubovaginal sling surgery offers long-term symptom improvement for patients requiring nonsynthetic sling placement.

Stress urinary incontinence (SUI) is a prevalent urological disorder among women, characterized by the involuntary leakage of urine during periods of cough, exertion, or physical activity^1^. This condition exhibits a wide prevalence across most nations, with high-risk factors including obesity, vaginal childbirth, and chronic coughing. The incidence rate approaches 40%, peaking at around 65% between the ages of 45 and 49^2^. The economic cost of treating and caring for SUI in women is substantial, and the consequential embarrassment stemming from urine leakage significantly impairs patients’ quality of life and social dynamics^3,4^. The diagnosis of SUI is predicated upon clinical physical examination and urodynamic assessment, which also serve as primary indicators for evaluating postoperative therapeutic efficacy.

The surgical treatment of SUI has evolved significantly over the past 30 years. Historically, the Burch colposuspension was the preferred choice for clinicians, and the trauma associated with the procedure was reduced with the advent of laparoscopic surgery. Since 1998, the mid-urethral suspension developed by Petros and Ulmsten^5^ has emerged as a leading procedure due to its minimal invasiveness and practical outcomes, rapidly becoming the standard operation worldwide and accounting for over 80% of SUI surgeries. The initial mid-urethral sling procedure involved the placement of a sling through the vagina at the mid-urethra and through two small incisions above the posterior pubic bone. In 2001, Delorme developed the transobturator tape (TOT), a sling placed outside through the obturator^6^. Subsequently, in 2003, de Leval reported placing the sling from the inside out through the obturator^7^. These two obturator pathways can reduce damage to the bladder, urethra, and surrounding vessels, but the postoperative incidence of groin pain is significantly higher than with the retropubic (RP) route. Recent years have witnessed the evolution of an innovative set of single-incision mini-slings. These are implanted through a vaginal suburethral cut and can either be attached to the obturator internus muscle, bypassing the involvement of the obturator fascia and muscles, or can be fastened retropubically to the urogenital diaphragm. Some urologists also perform autologous pubovaginal sling, particularly in patients who request nonsynthetic slings or those with a history of failed synthetic sling procedures.

Despite the variety of surgical options, numerous randomized controlled studies and meta-analyses have compared the efficacy of these procedures. The results indicate no significant difference in short-term effectiveness, including subjective and objective cure rates, between the RP and transobturator procedures^8–12^. Currently, literature is absent on long-term comparative effectiveness across diverse surgical strategies. This study, employing a network meta-analysis, executed both direct and indirect comparative statistical assessments on the outcome measures of various surgical methods and aimed to discern more efficacious interventions, thereby contributing robust evidence-based support for clinical decision-making.

## Materials and methods

The presentation of our results adhered to the Preferred Reporting Items for Systematic Reviews and Meta-Analysis (PRISMA, Supplemental Digital Content 1, http://links.lww.com/JS9/B236; Supplemental Digital Content 2, http://links.lww.com/JS9/B237)^13^, the extended network meta-analyses statement (PRISMA-NMA)^14,15^ and Assessing the Methodological Quality of Systematic Reviews (AMSTAR, Supplemental Digital Content 3, http://links.lww.com/JS9/B238) Guidelines^16^. As all data were extracted from open-source studies, the requirement for Institutional Review Board (IRB) approval was circumvented. In accordance with PROSPERO’s guidelines, registration of our study was undertaken and a CD number was obtained. Following completion of the study, update was appropriately made on the PROSPERO site^17^.

### Study inclusion and exclusion criteria

The studies will be included if it meets the following detailed inclusion criteria: all patients were adult females; all patients were diagnosed with SUI or mixed urinary incontinence, with SUI being the predominant symptom; interventions included all surgical treatment methods for SUI; outcomes: (i) Primary outcome: objective success rate, defined as the absence of leakage during the stress test or pad test. Subjective success rate, assessed by patient-reported questionnaires; (ii) Secondary outcomes: complications rate; reoperation rate; the duration of follow-up extends beyond 24 months; for identical outcome measures in the same study, data derived from the most extended follow-up interval is utilized; the type of study was randomized controlled trial, with no blinding requirement.

Studies with the following conditions were excluded: patients with serious comorbid organic diseases, such as neurological disorders and tumors; patients have severe neurological diseases that prevents normal participation in the experiment; data that was either unobtainable or not reported in a format that allows for statistical analysis; Non-English articles.

### Search strategy

A thorough and systematic literature review was undertaken across multiple databases, encompassing PubMed, EMBASE, MEDLINE, Cochrane Library, Medicine, and clinicaltrials.gov. Two reviewers independently executed the search process in May 2023. The search process was independently executed by two reviewers in May 2023. Based on the PICO framework and principles, the following search headings were used: ʻstress urinary incontinenceʼ, ʻstress incontinenceʼ, ʻurinary stress incontinenceʼ, ʻsurgeryʼ, ʻsurgical treatmentʼ, ʻBurchʼ, ʻcolposuspensionʼ, ʻTVTʼ, ʻtension-free vaginal tapeʼ, ʻTVT-Oʼ, ʻtension-free vaginal tape-obturatorʼ, ʻTOTʼ, ʻtransobturator tapeʼ, ʻsingle-incision mini slingʼ, ʻpubovaginal slingʼ, ʻurethral bulking agentsʼ. The search process involved the utilization of MeSH headings, free words, and keywords for searching, with the combination of searches achieved using Boolean operators (OR and AND).

### Study selection

Two independent reviewers implemented the literature selection process. When discrepancies arose, the intervention of a third reviewer was solicited to reach a consensus. Duplicate studies were identified and discarded during the initial screening performed with Endnote X9 software. Literature complying with the predefined inclusion criteria was subsequently shortlisted through the scrutiny of titles and abstracts. A subsequent screening process was instigated, during which literature adhering to the exclusion criteria was excised following an exhaustive review of the full text. Ultimately, the third author critically appraised studies that complied with the inclusion criteria.

### Data extraction

Data from the full text of the included articles was meticulously extracted by two independent reviewers using a predetermined form. Should discrepancies arise during the data extraction process, a resolution was attained either through a consensus between the two reviewers or with the intervention of a third reviewer. This form, constructed in accordance with the Cochrane Handbook guidelines^18^, encompassed various parameters: authorship, years of publication, demographic attributes of patients, implemented interventions, criteria for success, follow-up periods, and resultant outcomes. When a graphical representation of results was employed in the literature, the numerical transformation was facilitated by utilizing DigitizeIt software V2.0.

### Risk of bias assessment

The risk of bias (RoB) assessment within the included studies was carried out by adhering to the guidelines recommended by the Cochrane Collaboration tool^18^. Essential elements of this assessment encompass sequence generation, allocation concealment, blinding (or masking), evaluation of incomplete data, selective outcome reporting, and other potential bias sources. Consequently, the studies’ RoB was classified as ʻhighʼ, ʻunclearʼ, or ʻlowʼ. The bias assessment was independently executed by two reviewers, with studies generating disagreement being separately appraised by a third reviewer.

### Data synthesis

A Network Meta-Analysis (NMA) was undertaken within random-effects models utilizing Stata (Version 15.0) and R (Version 4.2.3). Mean differences (MD) and 95% CIs represented continuous variables, while risk ratios (RR) and 95% CIs conveyed dichotomous variables. The surface under the cumulative ranking curve (SUCRA) score was employed to rank the efficacy of each intervention and ascertain the optimal treatment. Interventions were rated based on their respective SUCRA values, with higher values indicating superior ranking.

Stata’s consistency model, incorporating the level of heterogeneity, the degree of inconsistency, and the magnitude of the treatment effect, facilitated the global consistency test. If the *P*-value was less than 0.05, the model’s inconsistency was deemed significant. The node-splitting approach was implemented for the local inconsistency test to identify potential inconsistency within a specific comparison.

## Results

**Figure 1:**
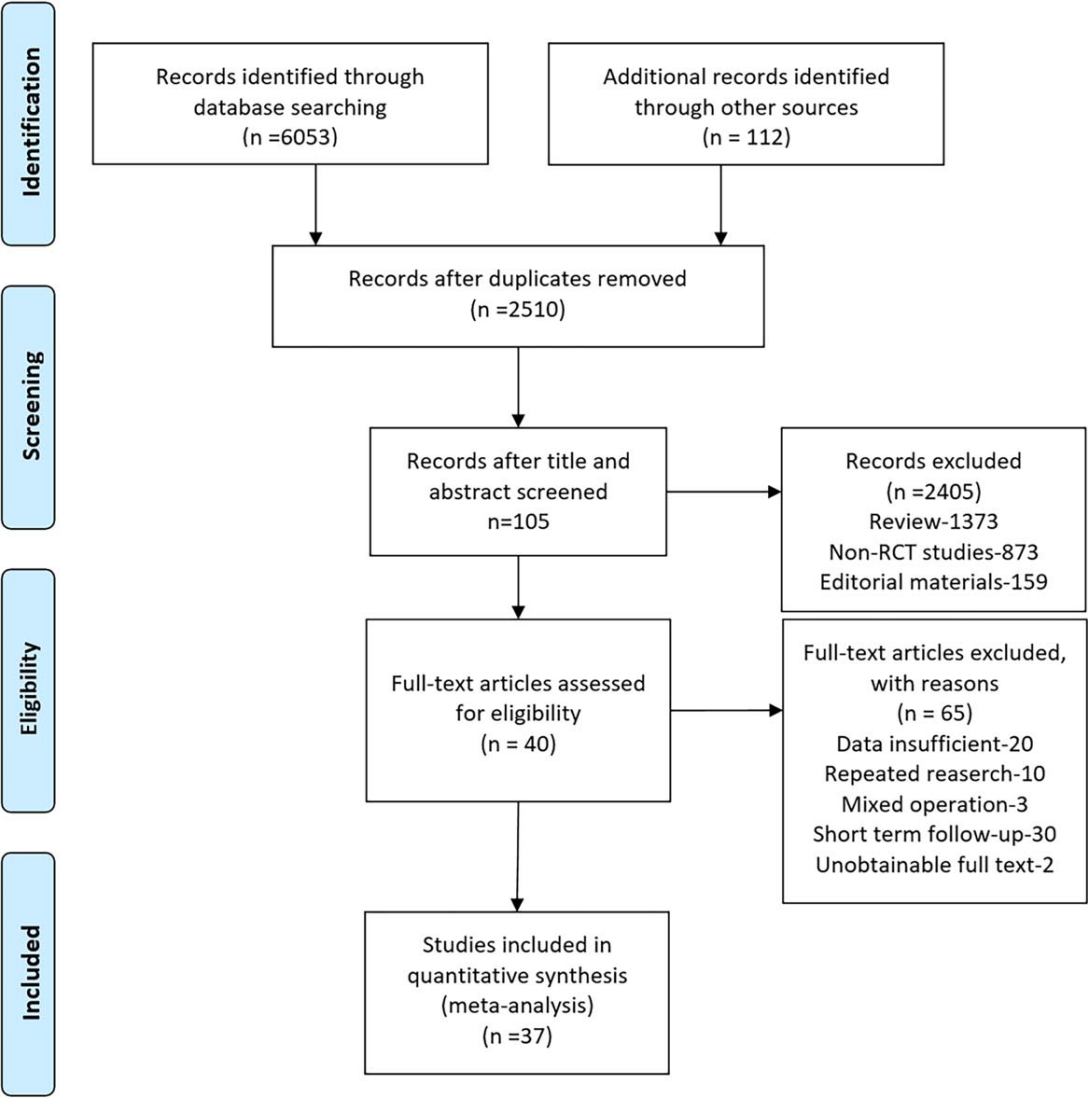
Flowchart of study search and selection.

In the 6053 articles retrieved, 37 randomized controlled trials^19–55^ with 5720 participants met the criteria and were included in the final statistical analysis (Fig. 1). Among the patients, the median age was 56.6 years (IQR 51–59), and the median follow-up period was 48 months (IQR 24–60). The information from the included literature is summarized in Table S1 (Supplemental Digital Content 5, http://links.lww.com/JS9/B240). The surgical methods for SUI reported in the included literature comprised retropubic tension-free vaginal tape (TVT-RP), tension-free vaginal tape-obturator (TVT-O), TOT, single-incision sling (SIS), Burch colposuspension, and pubovaginal sling (PVS). Other surgical methods were not included due to insufficient data preventing statistical analysis.

### Risk of bias assessment

The results of the risk of bias assessment revealed that the primary source of bias risk in the included literature originated from the blinding section, including the blinding of participants (35.1, 24.3, and 41.6%, respectively) and outcome assessment (37.8, 46.0, and 16.2%, respectively). Subsequently, risks related to incomplete outcome data bias (13.5, 21.6, and 64.9%, respectively) and allocation concealment (5.4, 21.6, and 73%, respectively) were identified. The risk associated with randomization (0, 2.7, and 97.3%, respectively) and selective reporting bias (0, 21.6, and 78.4%) in the included studies was relatively low (Figure S1, Supplemental Digital Content 4, http://links.lww.com/JS9/B239).

### Primary outcomes

**Figure 2:**
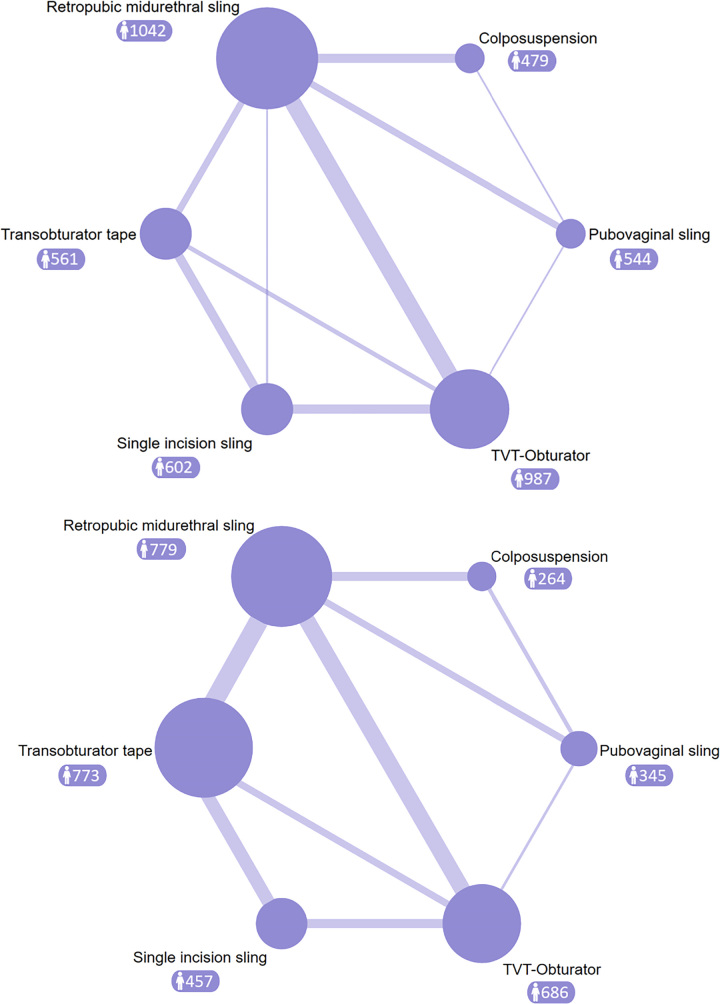
Network analysis of eligible comparison for (top) objective success rate and (bottom) subjective success rate. The size of each node represents the number of participants, while the thickness of the line represents the number of studies directly comparing the two interventions.

**Figure 3:**
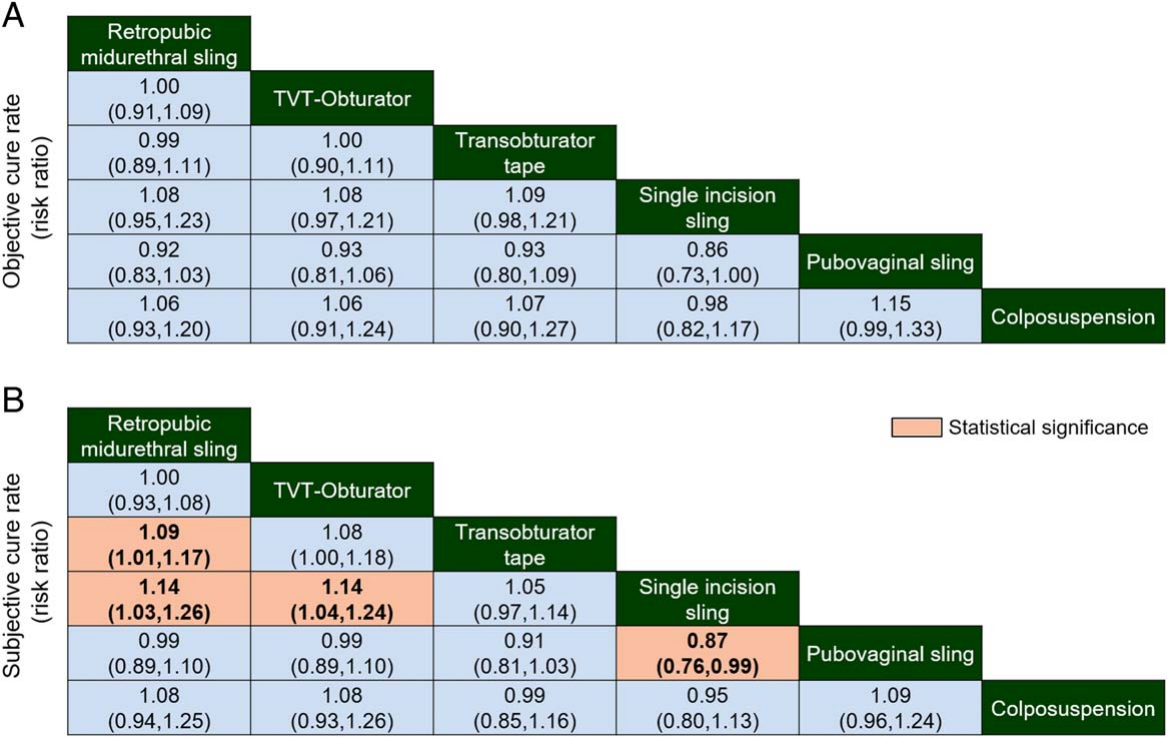
Comparative primary outcomes for (A) objective success rate and (B) subjective success rate. Significant results are in bold text.

All the included studies reported on the long-term follow-up outcomes for objective or subjective success rates following SUI surgery. Numerous studies have directly or indirectly compared the effects of TVT-RP and TVT-O surgical procedures (Fig. 2). Various surgical approaches exhibit comparisons among them, while there is a lack of direct comparative evidence between Burch colposuspension and SIS, as well as between PVS and SIS. The results of pairwise comparisons revealed no statistically significant differences among the various intervention measures in terms of postoperative objective success rates in patients. The comparative analysis of subjective success rates showed that TVT-RP demonstrated a slight superiority over TOT (1.09, 95% CI: 1.01–1.17) and SIS (1.14, 95% CI: 1.03–1.26), while TVT-O exhibited a marginal advantage over SIS (1.14, 95% CI: 1.04–1.24). SIS displayed a slightly lower success rate than PVS (0.87, 95% CI: 0.76–0.99). Pairwise comparisons for the remaining surgical procedures did not yield statistically significant differences (Fig. 3).

**Figure 4:**
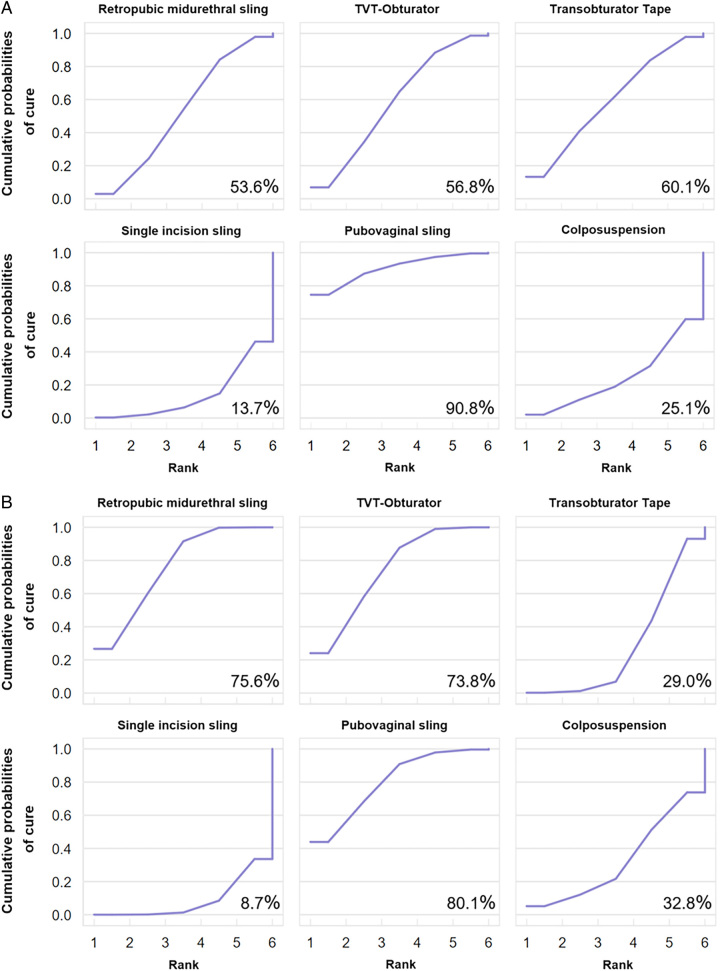
Surface under the cumulative ranking curve for (A) objective success rate and (B) subjective success rate. The vertical axis represents the probability for treatment to be the best option, the best of 2 options, the best of 3 options, and so on.

The ranking of long-term postoperative effectiveness for all intervention measures in terms of objective success rates indicated that PVS attained the highest SUCRA value (93.1), followed closely by TOT (60.1), TVT-O (56.8), and TVT-RP (53.6), while Burch (25.1) and SIS (13.1) occupied the lower positions in the ranking (Fig. 4). Regarding postoperative patient-reported success rates, PVS (80.1), TVT-RP (75.6), and TVT-O (73.8) rank highest with minimal differences. Following these, Burch colposuspension (32.8), TOT (29.0), and SIS (8.7) exhibited lower rankings. All outcome measures underwent both global and local consistency testing, with a consistency test *P*-value of 0.18 for objective success rates and a *P*-value of 0.60 for subjective success rates.

### Secondary outcomes

**Figure 5:**
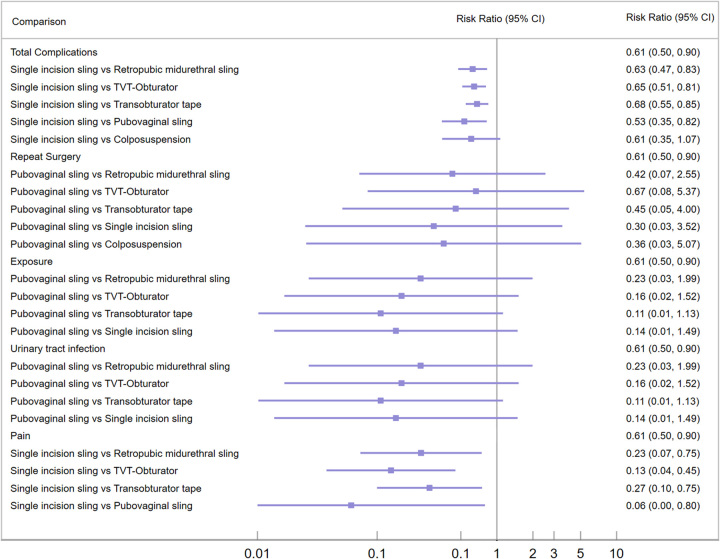
The forest plot presents comparative results of the overall incidence rate of complications, reoperation rate, exposure rate, incidence rate of urinary tract infections, and incidence rate of pain for different surgical techniques. Each outcome measure is compared to the reference group, which is determined by the highest surface under the cumulative ranking (SUCRA) value (indicating a lower incidence rate).

Each intervention with the highest SUCRA value for each outcome was selected as a reference to compare the overall complication rates, reoperation rates, and rates of various complications for each surgical procedure. The primary postoperative complications reported in the included studies were urinary tract infections, urinary retention, sling exposure, pain, and lower urinary tract symptoms, among which the data on the incidence of urinary retention and lower urinary tract symptoms were insufficient for statistical analysis. Compared to the other surgical procedures (except Burch colposuspension), SIS surgery had lower total complication rates and rates of pain occurrence, with statistical significance. There were no differences among the surgical procedures in terms of reoperation rate, sling exposure rate, and incidence of urinary tract infection (Fig. 5).

## Discussion

Surgical intervention for SUI has demonstrated beneficial effects, with significant alleviation of symptoms postoperatively. Since the introduction of mid-urethral sling procedures in the 1990s, these methods have gradually become the standard for SUI treatment due to their simplicity, effectiveness, and lower complication rates. Numerous clinical studies and reviews corroborate their efficacy and safety. Recent improvements in surgical techniques by urologists aim to identify even more effective and minimally invasive procedures. TVT-RP accounts for 43% of synthetic sling surgeries worldwide, with TO and TVT-O reaching 45% and SIS representing 12%^56^. Over 30 years since the commencement of sling surgeries, attention to their long-term efficacy has become increasingly important. Some studies have compared Burch, PVS, TVT-RP, and TOT, consistently finding no significant difference in the long-term effectiveness of TVT-RP and PVS, which outperform Burch surgery. However, the debate regarding the long-term efficacy between TVT-RP and TOT persists^14,57,58^. Hence, this study used a network meta-analysis approach to merge literature on the long-term efficacy of three generations of SUI surgeries. The findings indicate that, over a median follow-up period of 48 months, TVT-RP, TVT-O, and PVS demonstrate high subjective success rates, making them potentially preferable choices for treating female SUI. There are no significant differences among objective success rates among various surgical methods. Regarding safety, SIS, PVS, and Burch procedures have slightly lower long-term adverse event rates than other techniques. The results align with the short to medium-term follow-up results (12 months) of surgical treatment for SUI as outlined in the guidelines^59^. Therefore, for patients willing to undergo synthetic sling placement, the TVT-RP or TVT-O procedures can be considered, while for patients who do not wish to use synthetic slings or have experienced failure of synthetic sling surgery requiring reoperation, autologous PVS represents a long-term effective surgical approach.

The existing studies on the surgical treatment of SUI predominantly report short-term and mid-term follow-up results. Among these, the research on Burch and PVS surgeries is primarily early, with simple study designs, incomplete outcome measures, and low evidence levels. Studies comparing the efficacy of different surgeries using synthetic slings are numerous, with more comprehensive experimental methods, though the follow-up periods are mostly within 12 months. Long-term follow-up data focuses on objective success rates, subjective success rates, and complication occurrence rates. In these, the standard for objective success rate is relatively unified, defined as negative stress tests, while subjective success rates are evaluated based on various questionnaires and scales.

In the report of long-term complications, the primary complications include sling exposure, urinary tract infections, pain, urinary retention/difficulty urinating, and symptoms of overactive bladder. Sling exposure is a focal issue in postoperative complications. Symptoms caused by exposure to synthetic slings, including pain, urinary irritation, and urolithiasis, significantly affect the postoperative lives of patients. Meanwhile, negative publicity and strict policy supervision have posed challenges to sling surgery. This complication is related to the surgeon’s experience and the choice of puncture path. Novice surgeons are prone to visceral perforation and exposure. In 2017, the NHS England Mesh Oversight Group reported that sling application for SUI was safe, but evidence of long-term complications was lacking^60^. The results of this study show no statistically significant difference in the incidence of sling exposure between the various surgeries using synthetic slings. When compared with nonsynthetic sling surgery, there was no statistical difference.

This study still has certain limitations and constraints. Firstly, a significant limitation lies in the blind design adopted in most included literature. The implementation of blind methodologies is objectively restricted due to the inherent characteristics of the surgical procedure and cutaneous incisions, which could potentially influence the outcomes of follow-up assessments. Secondly, given the long-term nature of the follow-up data, a significant proportion of studies suffer from substantial loss to follow-up, leading to notable information bias. The patients who are successfully followed up often exhibit higher satisfaction with the surgery, potentially resulting in an overestimation of the surgical success rate. Some studies classify all lost-to-follow-up patients as surgical failures. In contrast, others disregard the resulting bias caused by this group, potentially inflating the final combined results of the surgical success rate. Furthermore, there is limited availability of long-term follow-up data for specific surgical procedures, such as bladder neck injections, which precludes conducting statistical analyses. Finally, the lack of direct comparative evidence between intervention measures also contributes to a decrease in the reliability of the results. For example, we still lack randomized controlled trials that provide direct comparison evidence between SIS and PVS and between SIS and Burch colposuspension.

## Conclusion

In long-term follow-up (median 48 months), TVT-RP, TVT-O, and PVS may be considered as surgical options that offer favorable effectiveness and safety profiles for the treatment of female SUI. SIS has lower overall complication and postoperative pain rates than other methods, while reoperation, sling exposure, and urinary tract infection rates do not significantly differ among the surgical approaches. TVT-RP and TVT-O are favorable options for patients willing to undergo synthetic sling placement, while autologous PVS surgery offers long-term symptom improvement for patients requiring nonsynthetic sling placement Table S2 (Supplemental Digital Content 6, http://links.lww.com/JS9/B323).

## Ethical approval

No application.

## Sources of funding

This study was supported by the National Key Research and Development Program of China (No. 2021YFC2009100). This study was funded by the National Natural Science Fund of China (Grant Nos. 8227031992).

## Author contribution

C.Y. and Z.C.: conception and design; D.-Y.L. and H.S..: administrative support; C.Y. and P.L.: provision of study materials or patients; C.Y., Z.C., Y.S., and C.J.: collection and assembly of data; C.Y. and Z.C.: data analysis and interpretation. All authors contributed in manuscript writing and final approval of manuscript.

## Conflicts of interest disclosure

The authors have no conflicts of interest to declare.

## Research registration unique identifying number (UIN)

1. Name of the registry: PROSPERO.

2. Unique identifying number or registration ID: CRD: 420234414353.

3. Hyperlink to your specific registration (must be publicly accessible and will be checked): https://www.crd.york.ac.uk/prospero/display_record.php?ID=CRD42023441435

## Guarantor

De-yi Luo, MD, PhD. Department of Urology, Institute of Urology, West China Hospital, Sichuan University; No. 37 Guo Xue Xiang, Chengdu, Sichuan 610041, People’s Republic of China. Tel: +86 189 8060 6809, fax: +86 28 8542 2451. E-mail: luodeyi1985@163.com; ORCID: 0000-0002-9436-036X

## Data availability statement

All data generated or analyzed during this study are obtained from published articles from known databases.

## Supplementary Material

SUPPLEMENTARY MATERIAL
