# Supplementary material for: Long-term outcomes of surgical interventions for stress urinary incontinence: a systematic review and network meta-analysis
Source: Int J Surg. 2023 Nov 2;110(1):520–8. doi: 10.1097/JS9.0000000000000828 (PMC10793800; doi:10.1097/JS9.0000000000000828)
Supplement: SUPPLEMENTARY MATERIAL [file js9-110-520-s001.doc]

Records identified through database searching

(n =6053)

**Screening**

**Included**

**Eligibility**

**Identification**

Additional records identified through other sources

(n = 112)

Records after duplicates removed

(n =2510)

Records after title and abstract screened

n=105

(n = 20 )

Records excluded

(n =2405)

Review-1373

Non-RCT studies-873

Editorial materials-159

Full-text articles assessed for eligibility

(n = 40)

Full-text articles excluded, with reasons

(n = 65)

Data insufficient-20

Repeated reaserch-10

Mixed operation-3

Short term follow-up-30

Unobtainable full text-2

Studies included in quantitative synthesis (meta-analysis)

(n =37)
