# Supplementary figures and images for: Long-term outcomes of surgical interventions for stress urinary incontinence: a systematic review and network meta-analysis
Source: Int J Surg. 2023 Nov 2;110(1):520–8. doi: 10.1097/JS9.0000000000000828 (PMC10793800; doi:10.1097/JS9.0000000000000828)

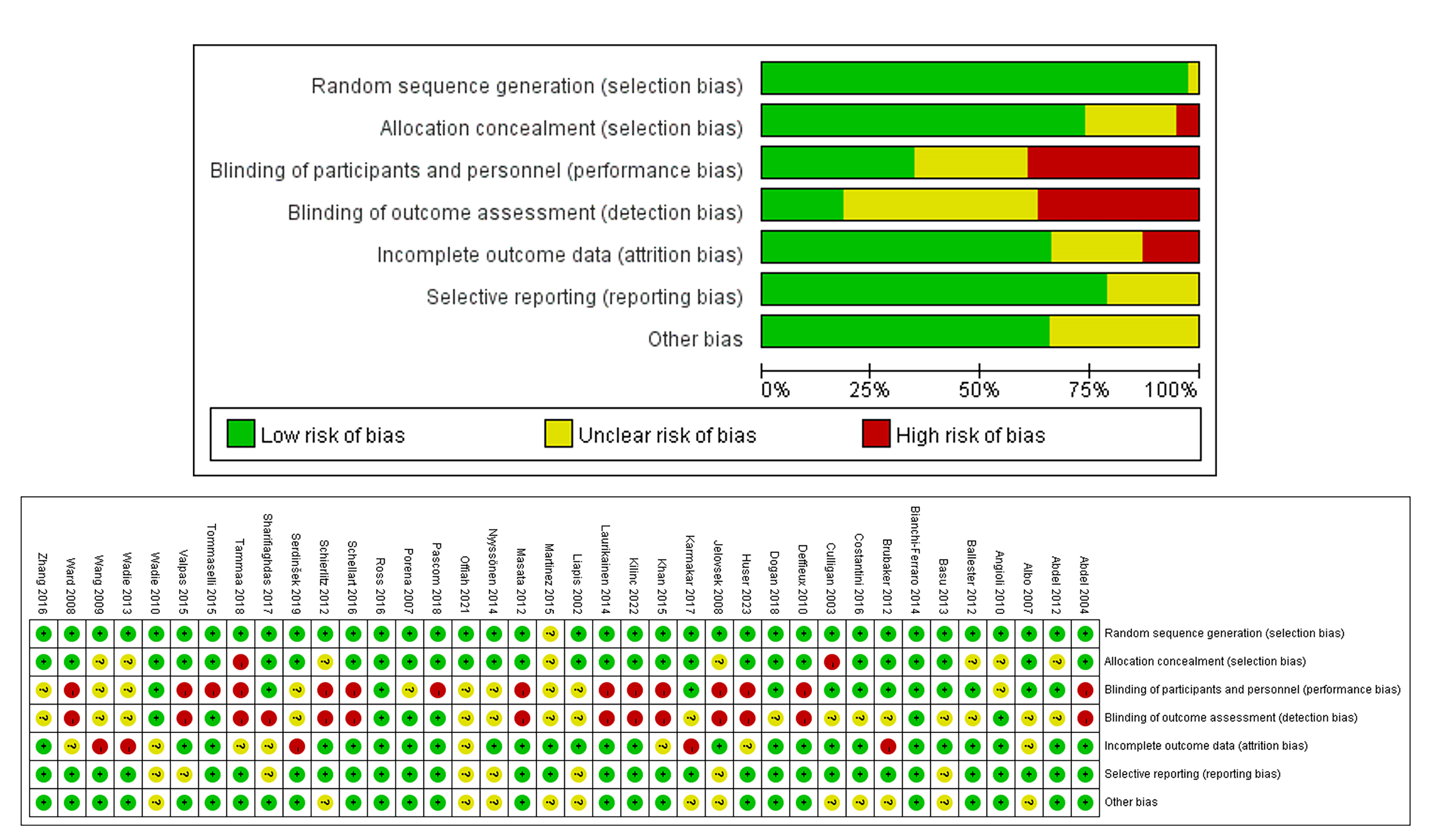

Supplement: SUPPLEMENTARY MATERIAL [file js9-110-520-s004.tiff]
