# Supplementary material for: Long-term outcomes of surgical interventions for stress urinary incontinence: a systematic review and network meta-analysis
Source: Int J Surg. 2023 Nov 2;110(1):520–8. doi: 10.1097/JS9.0000000000000828 (PMC10793800; doi:10.1097/JS9.0000000000000828)
Supplement: SUPPLEMENTARY MATERIAL [file js9-110-520-s005.doc]

**Table 1. Summary of included studies**

| Author, year | Trial Registration | Study design | Participants | Success criteria | Group samples | Follow-up period | Outcomes |
| --- | --- | --- | --- | --- | --- | --- | --- |
| Albo 2007 | NCT00064662 | Randomized,  Single-blind | Patients with pure or predominant symptoms of stress incontinence for at least 3 months and a positive standardized urinary stress test | Objective success: A negative stress test, and no retreatment for stress incontinence | 1. Modified colposuspension   (n=329)   1. Autologous pubovaginal sling   (n=326) | 24 months | Objective success rate, complications |
| Brubaker 2012 | NCT00064662 | Randomized,  Single-blind | Patients with pure or predominant symptoms of stress incontinence for at least 3 months and a positive standardized urinary stress test | Subjective success: Based on patient satisfaction questionnaire | 1. Modified colposuspension   (n=329)   1. Autologous pubovaginal sling   (n=326) | 60 months | Subjective success rate |
| Abdel 2004 | N/A | Randomized,  Non-blind | Not reported | Objective success: A negative stress test, and no retreatment for stress incontinence  Subjective success: Stress incontinence and quality of life improvement | 1. Pelvicol pubovaginal sling Bard   (n=72)   1. TVT-RP (Gynecare) (n=283) | 36 months | Objective success rate, complications |
| Abdel 2012 | NCT00136071 | Randomized,  Single-blind | Women with urodynamic SUI or mixed UI with a predominant SUI complaint who had previously failed or declined pelvic floor muscle treatment. | Subjective success: Very much improved/much improved on the PGI-I | 1. TVT-O (Ethicon Somerville, NJ, USA)   (n=147)   1. TOT (Coloplast Corp, Minneapolis, MN, USA)   (n=62) | 36 months | Subjective success rate, complications, KHQ, PISQ-12 |
| Angioli 2010 | NCT02757768 | Randomized,  Single-blind | Women with SUI have no  contraindications to vaginal surgery | Objective success: A negative stress test | 1. TVT-O (n=37) 2. TVT-RP (n=35) | 60 months | Objective success rate, complications, urodynamic parameters |
| Ballester 2012 | N/A | Randomized,  Single-blind | Women with SUI proven by clinical and urodynamic examinations | Objective success: A negative stress test | 1. TVT-RP (n=42) 2. TVT-O (n=195) | 48 months | Objective success rate, UDIQ, IIQ |
| Basu 2013 | N/A | Randomized,  Single-blind | Women with symptomatic and urodynamic SUI | Objective success: A negative stress test and no repeated surgery | 1. TVT-RP (Boston Scientific, Natick, MA, USA)   (n=33)   1. Single-incision sling (American Medical Systems, Minnetonka, MI, USA)   (n=38) | 36 months | Objective success rate, complications |
| Bianchi 2014 | NCT01095159 | Randomized,  Double-blind | Patients presenting SUI symptoms demonstrated by stress test and urodynamics | Objective success: A negative stress test  Subjective success: Absence of reports of urinary leakage indicated by a KHQ symptom scale score of 0 | 1. TVT-O (TVT-O™,Gynecare TVT Obturator System; Ethicon, Somerville, NJ, USA)   (n=56)   1. Single-incision sling (TVT-Secur, Gynecare TVT Secur System; Ethicon)   (n=66) | 24 months | Objective success rate, subjective success rate, complications, KHQ, |
| Culligan 2003 | N/A | Randomized | Women had urodynamic stress incontinence with urethral hypermobility | Objective success: A negative stress test  Subjective success: No leakage episodes reported during a standardized 24-h voiding diary. | 1. Colposuspension (n=15) 2. TVT-RP (n=13) | 72 months | Objective success rate, subjective success rate, urodynamic parameters |
| Costantini 2016 | N/A | Randomized,  Single-blind | Women with stress or mixed urinary incontinence associated with urethral hypermobility | Objective success: A negative stress test  Subjective success: Assessed with a 3-day voiding diary, quality-of-life questionnaires | 1. TVT-RP (Gynecare; Ethicon, Somerville, NJ, USA)   (n=47)   1. TOT (Mentor–Porges, Le lessis-Robinson)   (n=40) | ≥72 months | Objective success rate, subjective success rate, complications |
| Deffieux 2010 | NCT00135616 | Randomized,  Non-blind | Women with isolated SUI or mixed UI with a predominant SUI complaint | Objective success: A negative stress test  Subjective success: No referred leakage at interview, and no use of protection | 1. TVT-RP (Johnson and Johnson, Ethicon, Gynecare) (n=65) 2. TVT-O (Johnson and Johnson, Ethicon, Gynecare) (n=67) | 24 months | Objective success rate, subjective success rate, complications, urodynamic parameters |
| Dogan 2018 | IRB 000021705 | Randomized,  Single-blind | Women with SUI was diagnosed with clinical examination | Objective success: A negative stress test  Subjective success: No stress leakage of urine after surgery | 1. TOT (Contasure KIM® (Neomedic International) (n=89) 2. Single-incision sling (Contasure-Needleless® (Neomedic International) (n=89) | 24 months | Objective success rate, subjective success rate, complications |
| Huser 2023 | FNBr65269705 | Randomized,  Non-blind | Women with urodynamic SUI confirmed during cystometry by a positive standardized cough stress test | Objective success: A negative stress test  Subjective success: Very much improved/much improved on the PGI-I | 1. Single-incision sling Ophira (Promedon) surgical kit (n=66) 2. TOT (Gynecare Ethicon) (n=64) | 48 months | Objective success rate, subjective success rate, complications, ICIQ-SF, PPIUS |
| Jelovsek 2008 | N/A | Randomized,  Non-blind | Women had urodynamic SUI with abdominal leak point pressures greater than or equal to 60 cmH_2_O | Subjective success: No stress leakage of urine after surgery | 1. Colposuspension (n=28) 2. TVT-RP (Gynecare Inc, Somerville, NJ) (n=25) 3. Placebo (n=315) | 65 months | Subjective success rate, complications |
| Karmakar 2017 | N/A | Randomized,  Single-blind | Women with urodynamic SUI or stress predominant mixed UI, who failed or declined pelvic floor muscle training | Subjective success: Very much improved/much improved on the PGI-I | 1. TVT-O (Ethicon Inc, Somerville, NJ, USA) (n=104) 2. TOT (Coloplast Corp, Minneapolis, MN, USA) (n=104) | 108 months | Subjective success rate |
| Khan 2015 | NCT01057550 | Randomized,  Non-blind | Women (aged >18 years) with clinically and urodynamically confirmed SUI | Objective success: Women reporting being completely ‘dry’ or ‘improved’ at follow-up | 1. TVT-RP (Gynecare™,Somerville,NJ,USA) (n=63) 2. Autologous pubovaginal sling (n=61) | 120 months | Objective success rate, complications, HRQI, BFLUTS |
| Kilinc 2022 | NCT03949348 | Randomized,  Non-blind | Patients ≥18 years with SUI | Objective success: A negative stress test  Subjective success: No stress leakage of urine after surgery | 1. TVT-O (Ethicon women's Health) (n=60) 2. Autologous pubovaginal sling (n=60) | 24 months | Objective success rate, subjective success rate, complications, ICIQ-SF, I-QOL |
| Laurikainen 2014 | N/A | Randomized,  Non-blind | Women had a history of stress urinary incontinence and an indication for surgical treatment of their incontinence | Objective success: A negative stress test  Subjective success: Outcomes met patients’ expectations completely. | 1. TVT-RP (Gynecare, Ethicon, Johnson & Johnson, Somerville, NJ, USA) (n=131) 2. TVT-O (Gynecare, Ethicon, Johnson & Johnson, Somerville, NJ, USA) (n=123) | 60 months | Objective success rate, subjective success rate, complications, DIS, UISS, TBS, BFLUTS, PPIUSs |
| Liapis 2002 | N/A | Randomized | Women with SUI was diagnosed with clinical examination and urodynamics | Objective success: A pad weight difference <1 g | 1. Colposuspension (n=35) 2. TVT-RP (n=36) | 24 months | Objective success rate, complications, urodynamic parameters |
| Martinez 2015 | N/A | Randomized | Women affected by urodynamic SUI and a positive stress test | Objective success: A negative stress test | 1. TVT-O (n=108) 2. Single-incision sling (Contasure-Needleless® (Neomedic International) (n=131) | 54 months | Objective success rate, complications, ICIQ-SF |
| Masata 2012 | N/A | Randomized,  Non-blind | Women with proven urodynamic SUI | Objective success: A negative stress test  Subjective success: No stress leakage of urine after surgery, based on ICIQ-SF. | 1. TVT-O (Ethicon women's Health) (n=68) 2. Single-incision sling (TVT-Secur) (n=64) | 24 months | Objective success rate, subjective success rate, complications, ICIQ-SF, I-QOL, VAS |
| Nyyssönen 2014 | N/A | Randomized | Women with SUI or MUI with a predominant stress component | Subjective success: A postoperative UISS under 8 | 1. TVT-RP (Gynecare; Ethicon, Somerville, NJ, USA) (n=50) 2. TOT (Monarc, AMS solution; Minnetonka, Minnesota, USA) (n=50) | 46 months | Subjective success rate, UISS, DIS |
| Offiah 2021 | N/A | Randomized | Women over 21 years of age with SUI, or mixed urinary incontinence where SUI was the predominant symptom | Subjective success: Answering ‘no’ to question 8a on the ICIQ | 1. TVT-RP (Gynecare) (n=55) 2. TVT-O (Monarc, American Medical Systems (AMS)) (n=55) | 144 months | Subjective success rate, complications |
| Pascom 2018 | NCT01094353 | Randomized,  Single-blind | Women older than 18 years of age with SUI | Objective success: A negative stress test  Subjective success: Answering “very improved” or “improved” to question “how the patient felt about her incontinence problem after treatment.” | 1. TOT (Promedon,Argentina) (n=41) 2. Single-incision sling (n=41) | 36 months | Objective success rate, subjective success rate, complications, I-QOL, UDIQ |
| Ross 2016 | NCT00234754 | Randomized,  Double-blind | Women with symptomatic SUI based on clinical examination | Objective success: A negative stress test  Subjective success: Patients thought “urine loss was not reported as a big problem” | 1. TVT-RP (Boston Scientific; Natick, MA, USA)   (n=93)   1. TOT (Boston Scientific; Natick, MA, USA)   (n=83) | 60 months | Objective success rate, subjective success rate, complications, UDIQ, IIQ, PISQ-12 |
| Schellart 2016 | NTR3783 | Randomized,  Non-blind | Women with primary symptomatic SUI | Objective success: A negative stress test  Subjective success: Very much improved/much improved on the PGI-I | 1. Single-incision sling (MiniArc) (n=72) 2. TOT (TOT Monarc, American Medical Systems (AMS)) (n=69) | 24 months | Objective success rate, subjective success rate, complications, UDIQ, IIQ |
| Schierlitz 2012 | N/A | Randomized | Women had failed conservative management for SUI and who were diagnosed with intrinsic sphincter deficiency on urodynamic studies | Subjective success: Not requiring a repeat procedure on request of the patient | 1. TVT-RP (Gynecare, Ethicon, Inc, Somerville, NJ) (n=72) 2. TOT (American Medical Systems, Inc. Minnetonka, MN) (n=75) | 36 months | Objective success rate, reoperation rate |
| Serdinšek 2019 | N/A | Randomized | Women with SUI and also those  with MUI, where stress component was predominant | Objective success: A negative stress test  Subjective success: Absence of complaints about any involuntary leakage of urine on effort, exertion, sneezing, coughing or laughing | 1. TOT (Monarc) (n=53) 2. TVT-O (Ethicon,USA) (n=41) | 120 months | Objective success rate, subjective success rate, complications, UDIQ, IIQ, urodynamic parameters |
| Sharifiaghdas 2017 | N/A | Randomized,  Single-blind | Women had a history of USI, 1-hour pad test (more than 2-gram leak), objective positive cough | Objective success: A negative stress test  Subjective success: Based on the mean IIQ score in each group | 1. TVT-RP (n=37) 2. Autologous pubovaginal sling (n=32) | 126 months | Objective success rate, subjective success rate, complications |
| Tammaa 2018 | NCT00441454 | Randomized,  Open label | Women had a positive cough stress test at bladder filling of 300 mL without concomitant prolapse surgery or hysterectomy | Objective success: A negative stress test | 1. TVT-RP (Gynecare) (n=161) 2. TVT-O (Ethicon) (n=170) | 60 months | Objective success rate, complications, KHQ, VAS |
| Tommaselli 2015 | N/A | Randomized,  Single-blind | Women with SUI as diagnosed by clinical evaluation and urodynamics, age over 30 years, and previously failed pelvic floor muscle training | Objective success: A negative stress test  Subjective success: Very much improved/much improved on the PGI-I | 1. TVT-O (Ethicon Gynecare, Somerville, NJ) (n=62) 2. Single-incision sling (TVT-Secur Ethicon Gynecare, Somerville, NJ) (n=58) | 60 months | Objective success rate, subjective success rate, complications, I-QOL, |
| Valpas 2015 | N/A | Randomized,  Non-blind | Women with urodynamically  proven stress incontinence and a positive stress test | Objective success: A negative stress test  Subjective success: A significant decline in VAS and UISS scores compared with preoperative scores | 1. TVT-RP (Johnson & Johnson, Somerville, NY, USA) (n=70) 2. Colposuspension (n=51) | 60 months | Objective success rate, subjective success rate, UISS, VAS |
| Wadie 2013 | N/A | Randomized | Women had predominant stress incontinence with positive stress test | Objective success: A negative stress test | 1. TVT-RP (Gynecare, Ethicon) (n=36) 2. TOT (Coloplast) (n=35) | 24 months | Objective success rate, complications, UDIQ, IIQ |
| Wadie 2010 | N/A | Randomized,  Double-blind | Not reported | Objective success: A negative stress test | 1. TVT-RP (n=24) 2. Autologous pubovaginal sling (n=39) | 24 months | Objective success rate, complications, UDIQ, IIQ |
| Wang 2009 | N/A | Randomized | Women with demonstrable severe SUI, or mild or moderate SUI and failure of conservative therapy | Objective success: A negative stress test | 1. TVT-RP (Ethicon Inc, Somerville, NJ, USA) (n=154) 2. TVT-O (Ethicon Inc, Somerville, NJ, USA) (n=146) | 36 months | Objective success rate, complications |
| Ward 2008 | N/A | Randomized,  Non-blind | Women with urodynamic stress incontinence unresponsive to pelvic floor muscle exercise | Objective success: A negative stress test | 1. TVT-RP (Ethicon Inc, Somerville, NJ, USA) (n=72) 2. Colposuspension | 60 months | Objective success rate, complications, BFLUTS |
| Zhang 2016 | ChiCTR-TRC  -14004371 | Randomized | Patients who were diagnosed with SUI with urethral hypermobility and in whom conservative treatments had failed | Objective success: A negative stress test  Subjective success: Patient-reported cure/improvement | 1. TVT-RP (Ethicon Inc, Somerville, NJ, USA) (n=72) 2. TVT-O (Ethicon Inc, Somerville, NJ, USA) (n=49) | 95 months | Objective success rate, subjective success rate, complications |
| SUI: Stress Urinary Incontinence; MUI: Mixed Urinary Incontinence; TVT-RP: Retropubic Tension-free Vaginal Tape; TVT-O: Tension-free Vaginal Tape-obturator; TOT: Transobturator Tape; KHQ = King’s Health Questionnaire; PISQ-12: Prolapse and Incontinence Sexual Function Questionnaire-12; IIQ: Incontinence Impact Questionnaire; UDIQ: Urinary Distress Impact Questionnaire; ICIQ-SF: International Consultation of Incontinence Questionnaire-Short Form; I-QOL: Incontinence Quality of Life; PPIUS: Patient Perception of Intensity and Urgency Scale; HRQL: Health Related Quality-of-Life questionnaires; BFLUTS: Bristol Female Lower Urinary Tract Symptoms questionnaire; TBS: Treatment benefit scale; VAS: Visual Analog Scale; UISS: Urinary Incontinence Severity Score; DIS: Detrusor Instability Score; PGI-I: Patient Global Impression of Improvement. | | | | | | | |
